# Assessing the Implementation Determinants of Pilot Malaria Vaccination Programs in Ghana, Kenya, and Malawi through a Complexity Lens: A Rapid Review Using a Consolidated Framework for Implementation Research

**DOI:** 10.3390/vaccines12020111

**Published:** 2024-01-23

**Authors:** Abdu A. Adamu, Rabiu I. Jalo, Duduzile Ndwandwe, Charles S. Wiysonge

**Affiliations:** 1 Cochrane South Africa, South African Medical Research Council, Francie van Zijl Drive, Parrow Valley, Cape Town 7500, South Africa; duduzile.ndwandwe@mrc.ac.za (D.N.); sheyc@who.int (C.S.W.); 2 Division of Epidemiology and Biostatistics, Department of Global Health, Faculty of Medicine and Health Sciences, Stellenbosch University, Francie van Zijl Drive, Tygerberg, Cape Town 7505, South Africa; 3 Department of Community Medicine, Faculty of Clinical Sciences, Bayero University Kano, inside Aminu Kano Teaching Hospital, along Zaria Road, Kano 700233, Nigeria; rabiuibrahimjalo@yahoo.com; 4 Communicable and Non-Communicable Diseases Cluster, World Health Organization Regional Office for Africa, Djoue, Brazzaville BP 06, Congo

**Keywords:** malaria vaccine, RTS,S/AS01, implementation determinants, Ghana, Kenya, Malawi, systems thinking, consolidated framework for implementation research

## Abstract

In 2019, national immunization programs in Ghana, Kenya, and Malawi commenced the implementation of RTS,S/AS01 vaccination in large-scale pilot schemes. Understanding the implementation context of this malaria vaccination in the pilot countries can provide useful insights for enhancing implementation outcomes in new countries. There has not yet been a proper synthesis of the implementation determinants of malaria vaccination programs. A rapid review was conducted to identify the implementation determinants of the pilot malaria vaccination programs in Ghana, Kenya, and Malawi, and describe the mechanism by which these determinants interact with each other. A literature search was conducted in November 2023 in PubMed and Google Scholar to identify those studies that described the factors affecting malaria vaccine implementation in Ghana, Kenya, and Malawi. Thirteen studies conducted between 2021 and 2023 were included. A total of 62 implementation determinants of malaria vaccination across all five domains of the consolidated framework for implementation research (CFIR) were identified. A causal loop diagram showed that these factors are interconnected and interrelated, identifying nine reinforcing loops and two balancing loops. As additional countries in Africa prepare for a malaria vaccine roll-out, it is pertinent to ensure that they have access to adequate information about the implementation context of countries that are already implementing malaria vaccination programs so that they understand the potential barriers and facilitators. This information can be used to inform context-specific systems enhancement to maximize implementation success. Going forward, primary implementation studies that incorporate the causal loop diagram should be integrated into the malaria vaccine implementation program to enable immunization program managers and other key stakeholders to identify and respond to emerging implementation barriers in a timely and systematic manner, to improve overall implementation performance.

## 1. Introduction

Malaria is a mosquito-borne parasitic disease that is commonly spread in the tropical and sub-tropical regions of the world [1,2]. Although several species of the parasite exist, the most lethal among them is *Plasmodium falciparum* [2,3]. This disease has serious economic and public health consequences that impact the World Health Organization (WHO) African Region disproportionately [3,4]. The loss of productivity per year that is associated with malaria is estimated to be about USD 12 billion [5]. At the micro level, the cost of treating severe malaria pushes many households into poverty [6]. According to the World Malaria Report in 2022, there were an estimated 247 million cases of malaria, with 619,000 deaths in 2021, out of which 235 million cases (95%) and 593,000 deaths (96%) occurred in the African region [7]. In fact, four countries (Democratic Republic of the Congo, Mozambique, Nigeria, and Uganda) in Africa accounted for nearly 50% of all malaria cases in the world [7]. The majority of the malaria deaths recorded occurred in children [7]. Nevertheless, progress towards malaria elimination is advancing gradually as the number of malaria-endemic countries has been reduced from 108 in 2000 to 84 in 2021 [7]. This can be attributed to improvements in access to malaria diagnosis and case management, as well as preventive interventions such as insecticide-treated nets, indoor residual spraying, and seasonal malaria chemoprophylaxis [7]. Recently, malaria vaccination has also been added as a complementary preventive tool to further reduce the disease burden among children [8].

There are two malaria vaccines, RTS,S/AS01 and R12/Matrix-M, which have been recommended for use by the WHO [8,9]. The RTS,S/AS01 vaccine has already been deployed for use in real-world settings [10,11]. This is supported by evidence from a phase 3 clinical trial of the RTS,S/AS01 vaccine that was conducted in areas with varying malaria endemicity [12]. Recognizing its potential value, in 2016, the WHO recommended the vaccine’s pilot implementation in three African countries before country-wide introductions [13,14]. This pilot implementation project was coordinated by the WHO through the malaria vaccine implementation program (MVIP) [11]. The MVIP is a collaboration between the WHO, the ministries of health in the pilot countries, and their partners to generate additional evidence to support the widespread use of the vaccine [11].

The vaccine is to be administered in 4 doses to children aged 5 to 24 months who live in areas with moderate to high malaria endemicity [8]. The first dose of the vaccine should be administered at 5 months of age, after which a second and third dose should be provided, with a minimum of 4 weeks interval between each [8]; the fourth dose should be given after 12 to 18 months [8].

In 2019, national immunization programs in Ghana, Kenya, and Malawi commenced the implementation of RTS,S/AS01 vaccination in large-scale pilot schemes [15,16]. By the time the MVIP closed in December 2023, over 1.7 million children had been vaccinated with the RTS,S/AS01 vaccine in these countries [17]. Based on the successes recorded in the pilot countries, on 22 January 2024, Cameroon launched the first non-pilot malaria vaccination program in the world. Additional countries, including Burundi, Benin, Burkina Faso, Liberia, Niger, Sierra Leone, the Democratic Republic of the Congo, and Uganda will soon commence implementation through a phased scale-up [18]. However, understanding the implementation context in the pilot countries can enhance implementation in the new countries.

Growing evidence from the field of implementation science suggests that implementation efforts are prone to the effect of complex contextual factors; for this reason, health interventions can be successful in one setting and produce divergent results in other settings [19,20]. This is why it is important to thoroughly explore the contextual factors that affect implementation success in a specific setting where an intervention is being implemented, to have a good understanding of why this implementation succeeded or failed [19]. In the context of malaria vaccine introduction, the contextual determinants of implementation can provide important insights that will inform scale-up in Ghana, Kenya and Malawi, and to other countries as they allow proper planning and preparation to mitigate barriers and amplify facilitators that are similarly obtainable in those contexts [19].

A determinants framework can aid in the description of the contextual factors that influence the implementation of malaria vaccination programs in a robust manner to ensure the adequate consideration of broad external and internal elements [21]. The consolidated framework for implementation research (CFIR) is a well-validated determinants framework that has been widely used to evaluate health interventions [22,23]. The CFIR evaluates contextual factors across 5 domains, with 48 constructs and 19 sub-constructs [22]. The five domains are innovation, the inner setting, the outer setting, individuals, and the implementation process [22]. Innovation is the evidence-based intervention that is being implemented [22]. The inner setting refers to the place where the innovation is being implemented [22]. The outer setting is the environment that is external to the inner setting [22]. The term individuals refer to the people involved in implementing the innovation [22]. Finally, the implementation process comprises those activities or strategies that are deployed to implement the innovation [22].

Furthermore, the implementation determinants of malaria vaccination programs do not exist in isolation. In the real world, these determinants are connected to each other, and their interaction produces the system behavior that is observed. Therefore, systems thinking tools can be used to gain a better understanding of this complexity. There is an increasing consensus on the value of using systems thinking to explain the pathways and feedback in health services settings, given the complex adaptive nature of the health system [24,25]. The causal loop diagram (CLD) is an effective systems thinking tool for exploring the relationships between factors and solving problems using a complexity lens [26]. It is a qualitative tool for visually illustrating the linkages between factors to represent the system as a whole [27].

There is a dearth of research that uses an implementation science lens to study the contextual determinants of implementation regarding the pilot introduction of the RTS,S/AS01 malaria vaccine in Ghana, Kenya, and Malawi. This is important for immunization policymakers and other stakeholders at regional, national, and sub-national levels to guide planning, program design, and adaptation, especially in the new countries where the vaccine is being rolled out [19]. Therefore, the aim of this study was to identify the implementation determinants of the malaria vaccination program in Ghana, Kenya, and Malawi, and describe the mechanism by which these determinants interact with each other.

## 2. Methodology

A rapid review methodology was used for this research, in order to produce timely evidence for immunization program managers and policymakers at regional, national, and sub-national levels that can be used to guide preparations for the scale-up of the malaria vaccination program in new settings within the African region [28]. This approach to knowledge synthesis has emerged as a useful tool for fostering evidence-informed decision-making because it can be conducted within a shorter period of time compared to systematic reviews [29]. As additional countries in the African region prepare to introduce the malaria vaccine, there is a need for them to have a holistic understanding of “what worked” or “didn’t work” in settings where this vaccine has already been implemented, like Ghana, Kenya, and Malawi. For this reason, the review question that was chosen for this study is: “What are the implementation determinants that influenced malaria vaccine pilot introduction in Ghana, Kenya, and Malawi?”.

### 2.1. Search Strategy

A literature search was conducted on 29 November 2023 in two databases, PubMed and Google Scholar, to identify those studies that described factors affecting RTS,S/AS01 malaria vaccine implementation in Ghana, Kenya, and Malawi. A detailed search strategy was developed for the databases. These search strategies combined keywords using Boolean operators and applied truncations where necessary. In addition, when searching PubMed, medical subject headings (MeSH) and all fields were added to the keywords to further expand the search. The search strategy that was used is as follows: (“malaria vaccin*” OR “ RTS,S/AS01”) AND (barrier* OR constraint* OR bottleneck* OR limit* OR improv* OR facilitator* OR enable* OR drive* OR factor* OR understand* OR analys* OR challeng* OR implement* OR Introduc*). During the database search, no language restriction was specified. The database output was geographically restricted to Ghana, Kenya, and Malawi.

### 2.2. Inclusion and Exclusion Criteria

The sample, phenomenon of interest, design, evaluation, and research type (SPIDER) framework was used to guide the formulation of the eligibility criteria for this rapid review. The criteria for inclusion are as follows:

**Sample**: Studies conducted in Ghana, Kenya, and Malawi.

**Phenomenon of interest**: Studies conducted between 2019 and 2023 describing the barriers or facilitators of malaria vaccination with the RTS,S/AS01 vaccine.

**Design:** Observational studies

**Evaluation:** Studies that describe the perspectives and experiences of a broad range of stakeholders concerning malaria vaccination with RTS,S/AS01.

**Research type:** Mixed-methods, qualitative, and quantitative studies.

Studies were **excluded** if they were:

Focused on other types of malaria vaccines.

Conducted in settings outside of Ghana, Kenya, and Malawi.

### 2.3. Study Selection and Data Extraction

After removing any duplicates, two reviewers screened the titles and abstracts of 40% of the studies to identify those that were relevant. One reviewer screened the remaining studies while the second reviewer cross-checked those that were excluded. Then, the full texts of all the relevant studies were retrieved. One reviewer screened all the full texts for eligibility, based on the inclusion and exclusion criteria. The second reviewer cross-checked the excluded studies for correctness.

A data extraction tool that was developed in Microsoft Excel was used to collect all the relevant information from the included studies. The information that was extracted included: the author name, the country where the study was conducted, the study design, the study participants, and reported factors. One reviewer performed data extraction, while a second reviewer checked the data for completeness.

### 2.4. Narrative Synthesis Using Qualitative Analysis

A qualitative thematic analysis framework was used to describe the contextual determinants that influence the implementation of malaria vaccination in Ghana, Kenya, and Malawi [30]. This analytical framework is useful for identifying themes and patterns that are related to people’s personal experience of an intervention [31]. The factors extracted from individual studies were examined to make sense of the data and begin to organize them based on their relatedness. Upon refinement through an iterative process, the descriptive themes were inductively generated. When generating these themes, the linguistic reasoning of the primary studies was preserved as much as possible. To categorize these factors, each one of them was deductively mapped to the domains and constructs of CFIR. Factors that were related to the malaria vaccine itself were mapped to the innovation domain. Factors that were related to the healthcare facilities where the malaria vaccine is implemented were grouped under the inner setting domain. Factors that were related to the health system or society where the health facilities exist were classified under the outer setting domain. Factors related to the roles and characteristics of individuals were considered under the individual domain. Finally, factors related to those activities that were conducted to implement malaria vaccination were grouped under the implementation process domain. All the themes fit into the five domains.

### 2.5. Development of the Causal Loop Diagram

**Figure 1:**
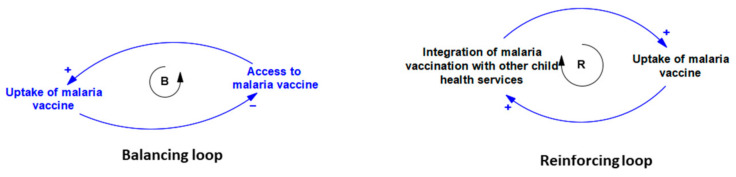
Examples of balancing and reinforcing loops.

To illustrate the dynamic relationship that exists between the implementation determinants of malaria vaccination programs in Ghana, Kenya, and Malawi, a causal loop diagram (CLD) was constructed. The CLD can show how the components of a system interrelate and the cause-and-effect linkages that exist between them [26]. In this study, the CLD is composed of the implementation determinants (variables) extracted from included studies and the linkages between them. The linkages, represented using arrows, show how the implementation determinants connect with each other and their direction of influence. The indicator of the influence is the polarity, which was denoted using (+) and (−) signs. If a determinant influences another determinant to change in the same direction, then a (+) sign was used. But if a determinant causes another determinant that it is linked with to change in the opposite direction, then a (−) sign was used. The feedback loops that were created through the interconnection between these implementation determinants are of two types: balancing (B) and reinforcing (R) feedback loops. In a balancing loop, the direction of change is countering, while in a reinforcing loop, the direction of change is compounding. Figure 1 is an example of a balancing and reinforcing loop. The balancing loop shows that access and uptake are connected. As access improves, uptake will increase, but an increase in uptake mops up the vaccines within the system, thereby making it unavailable. The reinforcing loop shows that there is a connection between service integration and updates of the vaccine. Integration of services leads to greater uptake of the vaccine. The established causal relationships were informed by the included studies and experiences of the authors. To validate the CLD, the authors reviewed the connections several times to ensure a practical representation of the system from their perspective.

## 3. Results

**Figure 2:**
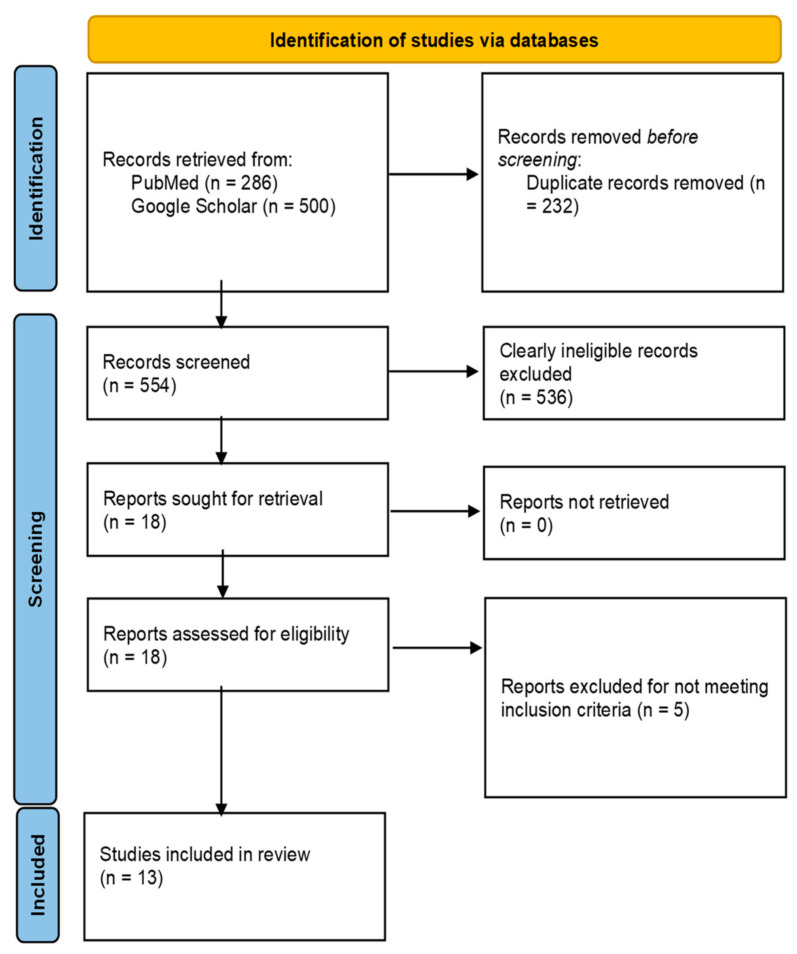
PRISMA flow diagram.

The total number of records that were found in Google Scholar and PubMed were 5720 and 286, respectively. However, only the first 500 results from Google Scholar were considered relevant. After screening and an eligibility assessment, a total of 13 publications were included in the study. The screening and selection process is presented in the PRISMA flow diagram shown in Figure 2.

### 3.1. Characteristics of Included Studies

The 13 reports that were included in this study concerned research conducted between 2021 and 2023 and covered all 3 countries, as shown in Table 1. Out of these studies, nine focused on Ghana alone. Different types of study designs were used, and they included qualitative (7 studies), quantitative (4 studies), and mixed-methods research (2 studies). The study covered diverse stakeholders, ranging from caregivers, health workers, and sub-national program implementers to national program implementers.

### 3.2. Implementation Determinants of Malaria Vaccine Pilot in Ghana, Kenya, and Malawi

The implementation determinants of malaria vaccination programs in Ghana, Kenya, and Malawi are multilevel, involving the vaccine itself, caregivers, health workers, health facilities, health systems, and society, as shown in Table 2. A total of 62 contextual implementation determinants were identified and they cut across all 5 CFIR domains, as follows: innovation (6 determinants), outer setting (8 determinants), inner setting (18 determinants), individuals (18 determinants), and the implementation process (12 determinants).

Table 3 shows how the contextual determinants that were identified as fitting into the CFIR constructs.

**Innovation domain:** This domain takes into account the determinants that are related to the malaria vaccine itself. The identified determinants matched with five constructs, which include innovation cost, evidence base, complexity, adaptability, and design.

**Inner setting domain:** This is the setting in which the malaria vaccine is being delivered, i.e., healthcare facilities. The identified determinants are linked with eight constructs that are related to the general features of the health facilities, as well as health facility characteristics that are specifically associated with vaccine delivery.

**Outer setting domain:** This domain reflects those factors that are external to the inner setting but that influence vaccine delivery, nonetheless. They originate from the health system and society. The identified factors aligned with five constructs in this domain, and they are as follows: critical incidents, local attitudes, local conditions, policies and laws, and financing.

**Individual domain:** The determinants within this domain reflect the characteristics of individuals that are involved in malaria vaccination. They include caregivers and health workers. Caregivers determine whether an eligible child will receive the vaccine, while health workers are the direct deliverers because they are responsible for administering the vaccine. The identified determinants are related to the following constructs: motivation, need, capability, and opportunity.

**Implementation process domain:** This domain represents the activities and strategies that were employed to implement the malaria vaccination program. The identified determinants are closely related to the following constructs: engaging, assessing context, team, adapting, performing, and tailoring the strategies.

**Figure 3:**
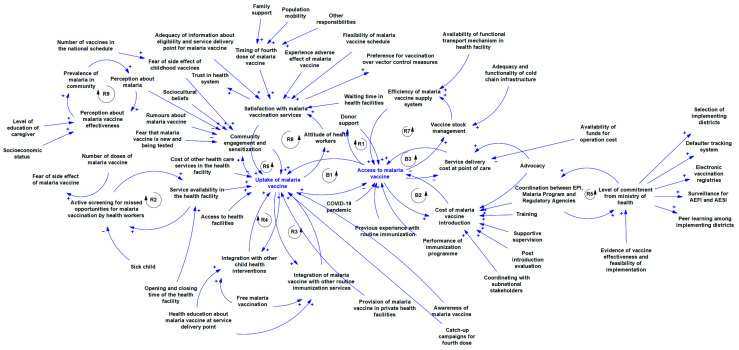
Causal loop diagram of implementation determinants of malaria vaccination programs in Ghana, Kenya, and Malawi, using data from the included studies.

The causal loop diagram in Figure 3 shows the interconnections and interrelatedness between multiple implementation determinants, illustrating the mechanism through which they influence implementation success. A total of nine reinforcing loops and three balancing loops were identified.

## 4. Discussion

This rapid review aimed to identify the implementation determinants of the malaria vaccination program in Ghana, Kenya, and Malawi, and describe the mechanism by which these determinants influence implementation success. After synthesizing the existing evidence, 62 implementation determinants were found across all 5 CFIR domains. In addition, it was found that dynamic linkages exist between these implementation determinants.

This study established that CFIR can be a useful theoretical framework to promote the effective implementation of malaria vaccination programs across different settings. CFIR was used to guide the data analysis and interpretation. In addition, it provided an opportunity to test the applicability and utility of CFIR in evaluating the implementation context of an immunization program. This enabled the identification of implementation determinants across domains that are clearly distinguishable, thus promoting a better understanding of the implementation context for malaria vaccination in Ghana, Kenya, and Malawi. The implementation context plays a critical role in determining whether an intervention that has been introduced in a particular place can be scaled up successfully [19]. This is why implementation science emphasizes the need for formative assessment to understand the implementation context in which an intervention like malaria vaccination is being implemented and to enable evidence-informed roll-out into other settings [45]. This study advanced the current literature by demonstrating the feasibility of using a rapid review methodology to analyze the implementation context and synthesize implementation determinants across different settings with moderate and high malaria transmission rates. The findings from this study can be used as a guide by countries that are about to commence the introduction of malaria vaccination programs, such as Cameroon, Burkina Faso, Liberia, Niger, Sierra Leone, the Democratic Republic of the Congo, and Uganda, to proactively plan and develop the relevant and appropriate approaches to maximize implementation success [18].

In this study, it was found that the drivers of implementation success of malaria vaccination programs are multiple and complex. This finding is similar to those obtainable for other health interventions and aligns with existing implementation science theories [21,46,47,48]. Determinants that are related to the malaria vaccine itself include cost, evidence base, complexity, adaptability, and design. Some are modifiable and can be planned for, while others, such as the number of doses and the timing of the fourth dose, are not because they impact the efficacy of the vaccine. It is unsurprising that cost was identified as vaccine introduction involves multiple activities like training, supervision, and meetings, all of which require funding. In addition, the service delivery cost, which includes health worker time, should be taken into account. The implication for this is that immunization programs should allocate adequate resources for these cost drivers to ensure successful implementation. If the age for vaccination is rigid, health workers will find it difficult to implement the program and coverage will likely remain low. There is a need to make the vaccination schedule as flexible as possible (guided by the evidence) and align it closely with routine immunization to increase the ease of delivery of all four doses at any healthcare contact point.

The majority of the determinants that were identified are related to the inner setting and individuals. The inner setting is responsible for delivering the vaccine; as such, the condition of this setting, in terms of structural characteristics, availability of resources, and the compatibility of malaria vaccination with existing functions, among others, plays an important role in influencing implementation success. Even if the inner setting is ready to deliver the vaccine, it is important to note that children are not “passive recipients” of the malaria vaccine [19]. The views and perceptions of their caregivers about vaccines in general, and the malaria vaccine in particular, their socioeconomic status, and their level of education, among several other factors, actively influence the decision to vaccinate. Therefore, it is important for countries that are preparing to roll out malaria vaccination programs to pay close attention to the inner setting and individuals in order to improve their chances of implementation success. The outer setting affects the inner setting and, to some extent, the individuals. For example, a critical incident like a disease outbreak can affect service provision as well as the movement of people, as seen with the COVID-19 pandemic [49,50]. Those activities that are conducted while implementing malaria vaccination are also crucial. Several activities that can influence implementation success have been identified in this study. One key example is community mobilization. The extent of community engagement and sensitization directly impacts implementation success. Moreover, this is a new vaccine and people are unfamiliar with it.

This study used a causal loop diagram to provide insight into how these contextual determinants interact to influence the implementation performance of malaria vaccination programs and the mechanism by which they affect access to and uptake of the vaccine. B1 is the access–uptake loop, which represents the foundational pillars of malaria vaccination because all implementation efforts are geared towards them. A major challenge with malaria vaccination is that the quantity of available vaccine doses is not sufficient to meet current demand. In fact, only 18 million doses of the vaccine have been allocated to these 12 African countries between 2023 and 2025 [51]. Bearing this in mind, the access–uptake feedback loop for the malaria vaccine shows that an increase in access increases uptake but as uptake increases the quantity of available vaccine will reduce thereby limiting access. Loops B2 and B3 show the relationship between access and the cost of vaccine introduction and service delivery cost at the point of care, respectively. Malaria vaccine introduction is a major cost driver as it involves multiple activities such as training, supervision, post-introduction evaluation, and coordination with sub-national stakeholders, as well as the malaria vaccination program. If facilities have sufficient overhead funds to cover the operation cost, the service delivery cost will be reduced.

Obviously, financial resources are needed to ensure the availability of the malaria vaccine. Loop R1 depicts the relationship between donor support and vaccine access. Loop R2 shows that if health workers are actively screening all children in the health facility for missed opportunities for malaria vaccination, then caregivers will become aware that services are available and, in turn, as caregivers become aware of service availability, they will be more receptive to active screening.

Both R3 and R4 are service integration loops. While R3 shows the feedback relationship of the incremental benefits of integrating malaria vaccination with routine childhood immunization services, R4 demonstrates the value of integrating vaccination with other child health services. Therefore, if malaria vaccination is available in a health facility, then it should be provided as part of an existing service rather than as a “stand-alone” intervention. Healthcare facilities should function as a “one-stop shop” for child health services, including immunization, to minimize the indirect cost of healthcare for caregivers. However, the success of integrating malaria vaccination with other routine childhood immunizations and other services depends on whether the vaccine is provided free of charge. An important determinant of implementation success for the malaria vaccination program that was identified in this analysis is service provision in private health facilities. It is important to bear in mind that some caregivers have a preference for private health facilities; therefore, the private health sector must be considered vital to ensuring equitable access.

The collaboration loop (R5) suggests that strong commitment from the Ministry of Health is needed to enhance close coordination between malaria control and immunization programs within the various countries. In fact, the impact of a strong commitment from the Ministry of Health has a specific programmatic advantage as it can spur increased investment in surveillance for adverse events following immunization and adverse events of special interest, a defaulter-tracking system, and the roll-out of electronic vaccination registers. However, the availability of evidence about the vaccine’s effectiveness and also about the feasibility of implementation of a malaria vaccination program seems to be an important motivating factor for ministries of health. Community engagement and sensitization are critical for stimulating the uptake of the vaccine, as shown by loop R6. Inadequate community engagement reduces the uptake of the malaria vaccine. When uptake is low, this means that only a few children in the community are given the vaccine, which reduces community awareness and chances for engagement. Several misconceptions exist about vaccines in general, and the malaria vaccine in particular, which can be addressed through context-appropriate engagement strategies. Loop R7 is the supply chain loop. Any delay or interruption to the supply of malaria vaccines to healthcare facilities will negatively affect access, and a decline in access will disrupt stock management, which will then impact forecasting and, invariably, supply. The last mile before delivery of the vaccine seems to be the most problematic as the unavailability of transport arrangements in health facilities to move the vaccines to those locations where they are required affects the vaccine supply. Also, if the vaccine cold-chain facility on the ground is not sufficient to accommodate the malaria vaccine, this will affect the ability of the program to ensure proper stock management. Loop R8 highlights the significance of the attitude of those health workers who offer malaria vaccination and also caregiver satisfaction with services. The poor attitude of health workers precipitates dissatisfaction with malaria vaccination services, which then decreases vaccine uptake. Loop R9 shows that the prevalence of malaria in a particular setting will influence the perceptions of people in that area regarding the malaria vaccine.

### Implications for Policy and Practice

**Figure 4:**
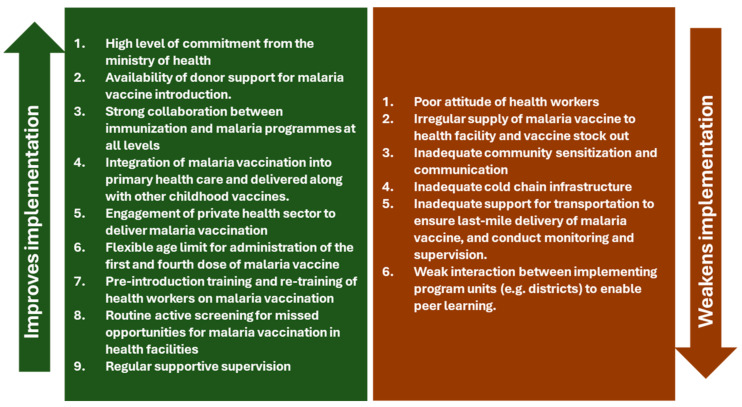
System levers for the implementation performance of malaria vaccination programs in Ghana, Kenya, and Malawi.

Based on the feedback in the causal loop diagram, some leverage points for systems changes or improvement were identified. These leverage points can be used to guide planning and preparation for vaccination roll-out in the new countries to optimize the implementation performance of their malaria vaccine programs. Figure 4 summarizes these system levers and delineates them into those points that improve or weaken implementation. It is important for country immunization teams to pay close attention to these system levers and institute the necessary actions to address any areas of weakness before roll-out.

This study has some limitations. Firstly, the causal loop diagram was constructed using secondary data, and, for this reason, some feedback might have been omitted. However, multiple data that were reported across different settings were included in the study, to improve the robustness and comprehensiveness of the causal statements. Secondly, there is a possibility of unconscious bias when developing the causal loop diagram. However, the authors that validated the linkages have previous experiences of immunization programs. Thirdly, most of the studies that were included in the review were conducted in Ghana; as such, the findings might be more representative of that context. Although contextual variations exist, the basic structure of the various national immunization programs is similar. Moreover, the intention of this study is to share lessons from an implementation context.

## 5. Conclusions

Substantial systems-wide pre-implementation planning improves the chances for the successful introduction of the malaria vaccine in a way that yields the expected outcome across diverse settings. Therefore, as additional countries in the African region prepare for malaria vaccine roll-out, it is pertinent to ensure that they have access to adequate information about the implementation context of countries that are already implementing malaria vaccination programs so that they understand the potential barriers and facilitators. This study filled this gap by applying systems thinking to evaluate the implementation determinants of malaria vaccination in Ghana, Kenya, and Malawi and unearthed multiple drivers of implementation performance, which interact in a complex manner. This provided a holistic understanding of the implementation context in these countries, which is useful for immunization stakeholders as they plan to scale up malaria vaccination programs in other African countries. This information can be used to inform context-specific systems enhancement and maximize implementation success. It is strongly recommended that primary implementation studies that incorporate CLD should be integrated into the malaria vaccine implementation program as it is scaled up to other countries, to enable immunization program managers and other key stakeholders to identify and respond to emerging implementation barriers in a timely and systematic manner to improve their overall implementation performance.

## Figures and Tables

**Table 1:** Characteristics of included studies.

S/No	Authors Name	Year of Publication	Country	Study Design	Target Population
1	Baral et al. [32]	2023	Ghana, Kenya, Malawi	Cross-sectional quantitative study	Government officials, health workers
2	Hoyt et al. [33]	2023	Kenya	Longitudinal qualitative study	Caregivers
3	Adeshina et al. [34]	2023	Ghana	Cross-sectional qualitative study	National program managers, research/academia, and program implementation partners
4	Okyere et al. [35]	2023	Ghana	Cross-sectional qualitative study	Health service providers and mothers
5	Merle et al. [36]	2023	Ghana, Kenya, Malawi	Cross-sectional qualitative study	Program managers
6	Adjei et al. [37]	2023	Ghana	Mixed methods study	EPI managers, coordinators and focal persons, healthcare workers, data managers, cold-chain managers, and caregivers
7	Bam et al. [38]	2023	Ghana	Cross-sectional qualitative study	Caregivers
8	Darkwa et al. [39]	2022	Ghana	Cross-sectional qualitative study	Caregivers of children that were involved in the RTSS pilot
9	Grant et al. [40]	2022	Ghana	Gross-sectional qualitative study	Regional and district health service managers and frontline health workers
10	Tabiri et al. [41]	2022	Ghana	Cross-sectional quantitative study	Caregivers
11	Yeboah et al. [42]	2022	Ghana	Cross-sectional quantitative study	Caregivers
12	Immurana et al. [43]	2022	Ghana	Cross-sectional quantitative study	Caregivers
13	Baral et al. [44]	2021	Ghana, Kenya, Malawi	Mixed-methods study	Ministry of Health officials at national and sub-national levels

**Table 2:** Contextual implementation determinants of malaria vaccination programs in Ghana, Kenya, and Malawi.

		Level of Influence					
S/No	Determinants	Malaria Vaccine	Caregiver	Health Workers	Health Facilities	Health System	Society
1	Cost of malaria vaccine introduction						
2	Service delivery cost at the point of care						
3	Satisfaction with malaria vaccination services						
4	Preference for vaccination over vector control measures						
5	Perception about malaria						
6	Free malaria vaccination						
7	Awareness of the malaria vaccine						
8	Previous experience with routine immunization						
9	Access to health facilities						
10	Perception about malaria vaccine effectiveness						
11	Attitude of health workers						
12	Adequacy of information about eligibility and the service delivery point for the malaria vaccine						
13	Fear of side effects of childhood vaccines						
14	Fear that the malaria vaccine is new and being tested						
15	Other responsibilities						
16	Service availability in the health facility						
17	Socioeconomic status						
18	Health education about malaria vaccine at service delivery point						
19	Active screening for missed opportunities for malaria vaccination by health workers						
20	COVID-19 pandemic						
21	Community engagement and sensitization						
22	Prevalence of malaria in the community						
23	Evidence of vaccine effectiveness and feasibility of implementation						
24	Availability of funds for operation cost						
25	Population mobility						
26	Number of vaccines in the national schedule						
27	Number of doses of malaria vaccine						
28	Family support						
29	Flexibility of malaria vaccine schedule						
30	Timing of the fourth dose of malaria vaccine						
31	Selection of implementing districts						
32	Fear of side effects of malaria vaccine						
33	Experience adverse effects from malaria vaccine						
34	Adequacy and functionality of cold-chain infrastructure						
35	Availability of functional transport mechanism in health facility						
36	Peer learning among implementing districts						
37	Efficiency of the malaria vaccine supply system						
38	Rumors about the malaria vaccine						
39	Training						
40	Level of commitment from the Ministry of Health						
41	Supportive supervision						
42	Post introduction evaluation						
43	Coordination between EPI, the malaria program, and regulatory agencies						
44	Coordinating with subnational stakeholders						
45	Integration with other child health interventions						
46	Defaulter tracking system						
47	Catch-up campaigns for the fourth dose						
48	Electronic vaccination registries						
49	Vaccine stock management						
50	Surveillance for AEFI and AESI						
51	Trust in the health system						
52	Waiting time in health facilities						
53	Advocacy						
54	Level of education of the caregiver						
55	Sociocultural beliefs						
56	Cost of other healthcare services in the health facility						
57	Opening and closing times of the health facility						
58	Sick child						
59	Performance of the immunization program						
60	Integration of the malaria vaccine into other routine immunization services						
61	Provision of the malaria vaccine in private health facilities						
62	Donor support						

CFIR—Consolidated framework for implementation research; level of influence—this is the system component that is related to the determinant. Color code: The colors represent the CFIR domains; the legend is provided below: 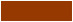
—Innovation; 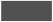
—Outer setting; 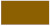
—Inner setting; 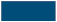
—Individuals; 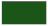
—Implementation process.

**Table 3:** CFIR constructs of the contextual determinants of malaria vaccine implementation performance in Ghana, Kenya, and Malawi.

CFIR Domains	CFIR Constructs	Identified Determinants
**Innovation**		
	Innovation cost	Cost of malaria vaccine introduction
	Innovation cost	Service delivery cost at the point of care
	Innovation evidence base	Evidence of vaccine effectiveness and the feasibility of implementation
	Innovation complexity	Number of doses of the malaria vaccine
	Innovation adaptability	Flexibility of the malaria vaccine schedule
	Innovation design	Timing of the fourth dose of malaria vaccine
**Outer setting**		
	Critical incidents	COVID-19 pandemic
	Local conditions	Prevalence of malaria in the community
	Local attitudes	Family support
	Policies and laws	Selection of implementing districts
	Local attitudes	Rumors about the malaria vaccine
	Local attitudes	Trust in the health system
	Local attitudes	Sociocultural beliefs
	Financing	Donor support
**Inner setting**		
	Relative priority	Level of commitment from the Ministry of Health
	Available resources	Free malaria vaccination
	Available resources	Availability of funds for operation cost
	Available resources	Adequacy and functionality of the cold-chain infrastructure
	Available resources	Availability of the functional transport mechanism in health facility
	Structural characteristics	Access to health facilities
	Structural characteristics	Service availability in the health facility
	Compatibility	Number of vaccines in the national schedule
	Access to knowledge and information	Health education about the malaria vaccine at the service delivery point
	Communication	Peer learning among the implementing districts
	Structural characteristics	Efficiency of the malaria vaccine supply system
	Access to knowledge and information	Training
	Structural characteristics	Waiting times in health facilities
	Compatibility	Cost of other healthcare services in the health facility
	Structural characteristics	Opening and closing times of the health facility
	Structural characteristics	Performance of the immunization program
	Structural characteristics	Integration of the malaria vaccine with other routine immunization services
	Relational connections	Provision of the malaria vaccine in private health facilities
**Individuals**		
	Capability	Active screening for missed opportunities for malaria vaccination by health workers
	Capability	Attitudes of health workers
	Motivation	Satisfaction with malaria vaccination services
	Need	Preference for vaccination over vector control measures
	Need	Perceptions about malaria
	Need	Awareness of the malaria vaccine
	Need	Previous experience with routine immunization
	Need	Perception about malaria vaccine effectiveness
	Opportunity	Adequacy of information about eligibility and service delivery points for the malaria vaccine
	Need	Fear of side effects of childhood vaccines
	Need	Fear that the malaria vaccine is new and being tested
	Need	Sick child
	Opportunity	Other responsibilities
	Opportunity	Socioeconomic status
	Opportunity	Population mobility
	Need	Fear of side effects of the malaria vaccine
	Motivation	Childexperienced adverse effects from the malaria vaccine
	Capability	Level of education of the caregiver
**Implementation process**		
	Engaging	Community engagement and sensitization
	Engaging	Supportive supervision
	Assessing context	Post-introduction evaluation
	Teaming	Coordination between the EPI, the malaria control program, and regulatory agencies
	Teaming	Coordinating with subnational stakeholders
	Adapting	Integration with other child health interventions
	Performing	Defaulter tracking system
	Performing	Catch-up campaigns for the fourth dose
	Tailoring strategies	Electronic vaccination registries
	Performing	Vaccine stock management
	Assessing context	Surveillance for the AEFI and AESI
	Engaging	Advocacy

CFIR—consolidated framework for implementation research; EPI—expanded program on immunization; AEFI—adverse events following immunization; AESI—adverse events of special interest.
